# Comparable rates of secondary surgery between anterior cruciate ligament repair with suture tape augmentation and anterior cruciate ligament reconstruction

**DOI:** 10.1186/s40634-022-00549-w

**Published:** 2022-12-02

**Authors:** G. P. Hopper, W. T. Wilson, L. O’Donnell, C. Hamilton, M. J. G. Blyth, G. M. MacKay

**Affiliations:** 1 NHS Lanarkshire University Hospitals, 218 Eaglesham Road, East Kilbride, Glasgow, Scotland G75 8RG UK; 2 Rosshall Hospital, Glasgow, UK; 3 NHS Greater Glasgow & Clyde, Glasgow, UK

## Abstract

**Purpose:**

The aim of this study was to determine the frequency of secondary surgery following anterior cruciate ligament (ACL) repair with suture tape augmentation in comparison to conventional hamstring ACL reconstruction. We hypothesised that there would be no differences between the groups.

**Methods:**

This was a retrospective comparison study of patients undergoing ACL surgery between September 2011 and April 2018. Two hundred and 73 patients underwent ACL reconstruction using hamstring autograft. During the same timeframe, 137 patients with an acute proximal ACL rupture underwent ACL repair with suture tape augmentation. One patient was lost to follow-up in the ACL reconstruction group leaving 272 patients (99.6%) for the final analysis. In the ACL repair group, three patients were lost to follow-up leaving 134 patents (97.8%) for the final analysis. Secondary surgery was identified by contacting the patients by email/telephone and reviewing patient notes at the time of this analysis.

**Results:**

Re-rupture occurred in 32 patients (11.8%) in the ACL reconstruction group compared to 22 patients (16.4%) in the ACL repair group (*p* = 0.194). Contralateral ACL rupture occurred in four patients (1.5%) in the ACL reconstruction group compared to three patients (2.2%) in the ACL repair group (*p* = 0.224). In the ACL reconstruction group, nine patients (3.3%) required secondary meniscal surgery whilst five patients (3.7%) required meniscal surgery in the ACL repair group (*p* = 0.830). Seven other operations were performed in the ACL reconstruction group (2.6%) compared to three other operations in the ACL repair group (2.2%) (*p* = 0.374). The overall number of patients undergoing secondary surgery in the ACL reconstruction group was 52 (19.1%) in comparison to 30 (22.4%) in the ACL repair group (*p* = 0.114).

**Conclusion:**

ACL repair with suture tape augmentation for acute proximal ruptures demonstrated comparable rates of secondary surgery with hamstring ACL reconstruction.

## Introduction

Although ACL reconstruction has been the gold standard treatment for the past 30 years, both ipsilateral graft rupture and contralateral ACL injury are worrying complications for patients [11, 12, 16, 22]. A systematic review by Wiggins et al. [22] showed that following reconstruction, the secondary ACL reinjury rate (ipsilateral and contralateral re-injury combined) was 21% for patients under 25 years old. Ipsilateral re-injury rates vary in the literature but have been reported to be as high as 33% in young patients [20, 21]. Indeed, Paterno et al. [15] showed that 21% of young patients who have ACL reconstruction, subsequently have a contralateral ACL injury.

Further surgery for meniscal tears following ACL reconstruction has also been reported to be as high as 12% [18]. Other reasons for re-intervention may include complications such as infection, stiffness or hardware irritation. All of these reasons combined make the rates of further knee surgery after ACL reconstruction alarmingly high. Indeed, from the large MOON group series, the authors demonstrated a 29% rate of further knee surgery within 6 years of ACL reconstruction [6].

An enhanced understanding of ACL healing, advancements in techniques and concerns over the complications associated with ACL reconstruction has resulted in a renewed interest in ACL repair [19]. ACL repair with suture tape augmentation reinforces the ligament and encourages natural healing by protecting it during the healing phase and supporting early mobilisation [23]. This technique avoids the need for graft harvest and retains the proprioceptive fibres of the native ACL. Other contemporary ACL repair techniques have recently been described in the literature including dynamic intraligamentary stabilization (DIS) which uses a device with an internal dynamic screw-spring mechanism that stabilizes the knee during the healing phase. However, this technique has been associated with an extremely high, 48% reintervention rate [5]. Some evidence is emerging regarding rates of ipsilateral failure of ACL repair surgery, but there is limited information on combined secondary surgery rates [1–4, 7–10, 17]. This study aimed to determine the incidence of secondary surgery following ACL repair with suture tape augmentation in comparison to conventional hamstring ACL reconstructions. We hypothesized that there would be no differences between the two groups.

## Methods

### Study design and participants

Ethical approval for the study was granted by the University of Strathclyde (UEC19/24). This study was a retrospective comparison study of patients undergoing ACL surgery between September 2011 and April 2018. Two hundred and 73 patients with an ACL rupture underwent ACL reconstruction using hamstring autograft. In the same timeframe, 137 patients with an acute proximal ACL rupture underwent ACL repair with suture tape augmentation. All operations were performed by the two senior authors (MJGB and GMM). Inclusion criteria for ACL repair were acute proximal tears with adequate ACL tissue quality within 3 months of injury. Patients with mid-substance, distal ACL ruptures and retracted ACL remnants were not suitable for ACL repair. Patients who had multiligament injuries or surgery to the anterolateral ligament were excluded from the study.

There was one patient lost to follow-up in the ACL reconstruction group leaving 272 patients (99.6%) for the final analysis. In the ACL repair group, three patients were lost to follow-up leaving 134 patents (97.8%) for the final analysis.

### Surgical technique

The ACL reconstruction technique was a four-strand semitendinosus-gracilis graft in the anteromedial femoral position, secured with a femoral Endobutton (Smith & Nephew) and a tibial Intrafix interference screw and sheath (DePuy).

**Fig. 1:**
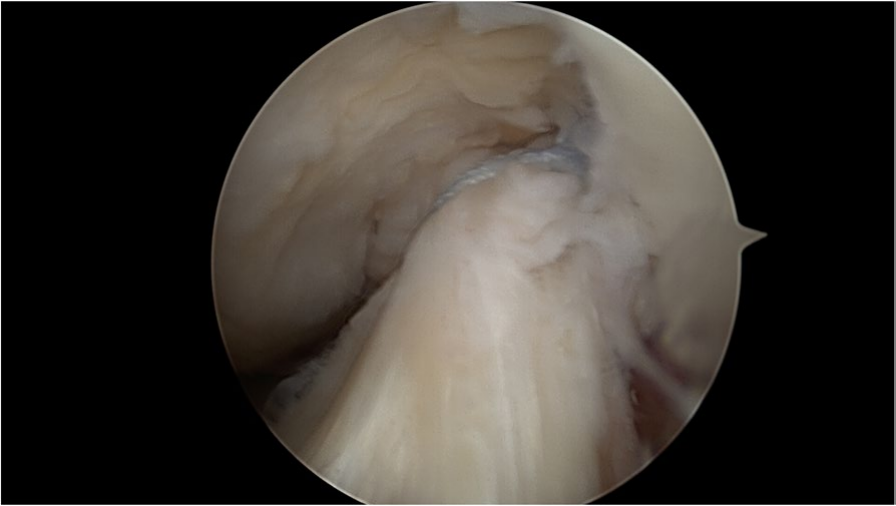
Arthroscopic image of a repaired ACL

Proximal ruptures of the ACL were repaired with suture tape augmentation (FiberTape, Arthrex) after assessing suitability with a probe. The ACL remnant was left intact and 3.5 mm tibial and femoral tunnels were drilled. A femoral button (TightRope, Arthrex) loaded with suture tape and a 4.75 mm tibial anchor (SwiveLock, Arthrex) were used to secure the repair. (Fig. 1).

### Secondary surgery assessment

Secondary surgery was identified by contacting the patients by email/telephone as well as reviewing patient notes at the time of this analysis. Information recorded included basic demographics, ipsilateral graft rupture, contralateral ACL rupture, secondary meniscal surgery and any other further surgery on the ipsilateral or contralateral knee.

### Statistical analysis

Descriptive statistics were calculated to summarise the demographics and clinical characteristics and described with means and standard deviations for normally distributed data or median with ranges for non-normally distributed data. Normality was assessed using the Shapiro–Wilk test. Unpaired students t-tests or Mann-Whitney U tests were performed to compare variables between groups. Chi-squared tests used to identify differences in occurrence rates between groups and the significance level set a *p* < 0.05. A power calculation post hoc was performed but in order to achieve 80% power in this study, a sample size of over 3000 patients would be required. All analyses were performed with SPSS, version 27 (IMB, Chicago, IL, USA).

## Results

**Fig. 2:**
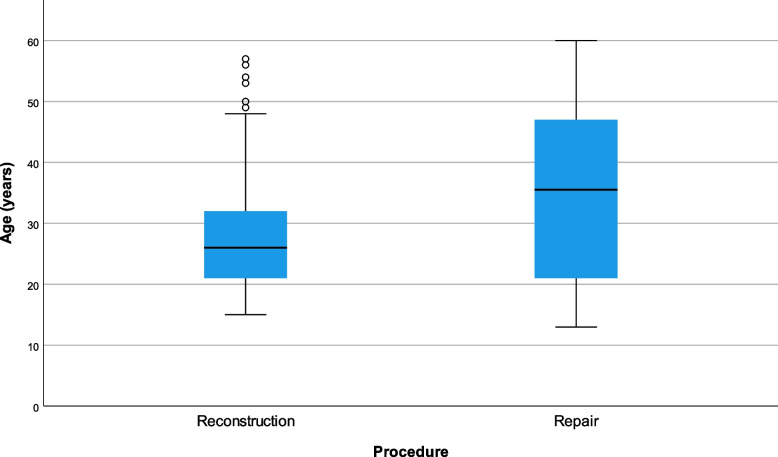
Boxplot demonstrating the younger age of the ACL reconstruction group compared to the ACL repair group

The mean age of the ACL reconstruction group was lower, 28 (±9) years, than the ACL repair group, 35 (±14) years (*p* < 0.01). (Fig. 2) There was no difference in follow-up time, which was a mean of 5 years for both groups, with minimum follow-up period of 2 years postoperatively. The median interval from injury to surgery was significantly longer in the ACL reconstruction group (12 months v 1 month, *p* < 0.001).

There were a higher proportion of males in the ACL reconstruction group (82% v 56%, *p* < 0.01). Pre-injury activity level was the same in both groups, with a median Tegner score of seven. Most injuries were sustained during sport with football being the most common overall. Football was more heavily represented as a mechanism of injury in the ACL reconstruction group (61%) than for ACL repair (32%), while skiing was more prevalent for those undergoing ACL repair (40%) than ACL reconstruction (4%).

**Fig. 3:**
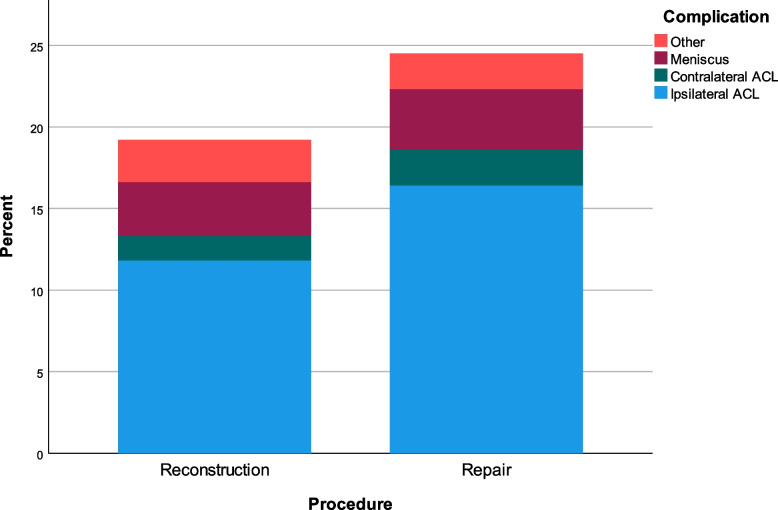
Stacked bar chart illustrating the reasons for secondary surgery following ACL reconstruction and ACL repair

Ipsilateral re-rupture occurred in 32 patients (11.8%) in the ACL reconstruction group compared to 22 patients (16.4%) in the ACL repair group (*p* = 0.194) (Table 1). Contralateral ACL rupture occurred in four patients (1.5%) in the ACL reconstruction group compared to three patients (2.2%) in the ACL repair group (*p* = 0.224) (Fig. 3). These totals excluded 13 patients in the ACL reconstruction group and two patients in the ACL repair group who had pre-existing contralateral ACL injuries.

**Table 1:**
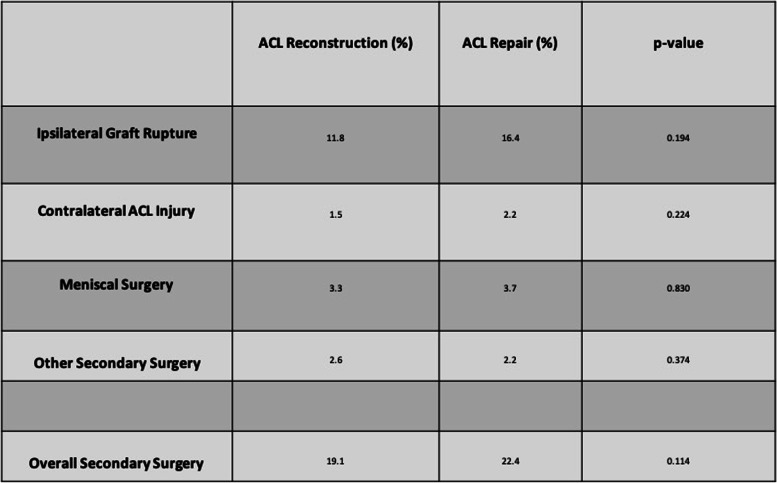
Secondary surgery rates for both ACL reconstruction and ACL repair cohorts and *p*-values from Chi-squared tests demonstrating no significant differences.

In the ACL reconstruction group, nine patients (3.3%) required subsequent meniscal surgery, which consisted of four ipsilateral partial medial meniscectomies, one ipsilateral partial medial and lateral meniscectomy and four contralateral meniscectomies. In comparison, five patients (3.7%) required meniscal surgery in the ACL repair group consisting of three ipsilateral partial medial meniscectomies and two ipsilateral partial lateral meniscectomies with no contralateral surgical procedures (*p* = 0.830).

Other secondary surgery in the ACL reconstruction group (2.6%) included three washouts for infection, two chondroplasties, one manipulation under anaesthetic for stiffness and one total knee replacement. Other secondary surgery in the ACL repair group (2.2%) included two manipulations under anaesthetic for stiffness and one chondroplasty (*p* = 0.374).

The overall number of patients undergoing secondary surgery in the ACL reconstruction group was 52 (19.1%) in comparison to 30 (22.4%) in the ACL repair group (*p* = 0.114). In some instances, more than one procedure was carried out during the same surgical episode, for example meniscal surgery in conjunction with an ACL procedure.

## Discussion

The results of this study demonstrate rates of secondary surgery in patients undergoing ACL reconstruction similar to those reported in other studies [11, 12, 16, 22]. Moreover, rates of secondary surgery for ACL repair with suture tape augmentation are not significantly different, confirming the null hypothesis. These rates are considerably superior to the 48% reintervention rate described for DIS ACL repair [5]. This would suggest that ACL repair with suture tape augmentation is a safer technique with lower reintervention rates. This has the benefit of preventing potential further morbidity, time out of sport or employment, risks of further anaesthetic as well as the financial burden.

Indeed, recent literature has demonstrated encouraging results with ACL repair with suture tape augmentation. Jonkergouw et al. demonstrated good objective and subjective outcomes at 3.2-year follow-up of 56 ACL repairs which included 27 patients with suture tape augmentation [10]. Another study demonstrated good functional outcomes and a low re-rupture rate of 3%, however, this study was based on minimum 12-month follow-up [17]. Furthermore, Douoguih et al. revealed a 14.8% re-rupture rate in their study of 27 patients with minimum 2-year follow-up and a similar study showed a rate of 6.9% in 29 patients [1, 3]. Recently, a five-year follow-up prospective evaluation revealed a 17.6% re-rupture rate and they found those patients to be younger and have significantly higher activity levels than the rest of the cohort [9]. They also published a further study which revealed that those high-risk patients benefit from an additional ALL procedure, however, this was at short-term follow-up [8]. Conversely, Gagliardi et al. demonstrated a high failure rate in adolescents undergoing ACL repair [4].

Despite the reintervention rates for both procedures in this study being lower at mean 5 years follow-up than some reports in the literature [6, 21], it remains that around one in five patients will have further knee surgery in that short time frame. This is despite the latest advances in arthroscopic surgery and postoperative rehabilitation. Patients undergoing primary ACL surgery of any kind should be counselled appropriately.

The rate of contralateral ACL rupture after return to sport is approximately 2% in our cohorts, which is lower than the rates quoted for ACL reconstruction in the literature [13–15]. This may be explained as we do not know the rate of return to sport in this study for the ACL reconstruction cohort, a limitation of its design. Several factors including socio-economic and psychological mean that patients may never return to sport following ACL reconstruction. For the ACL repair cohort, we know that 87% returned to sport, yet the contralateral injury rate remains low. This may be a consequence of retained proprioception from preserved native ACL tissue, which in turn may improve functional performance of the knee pre-disposing less to contralateral injury. Reassuringly, there was a low rate of intervention for postoperative stiffness (2.2%) in the ACL repair group, addressing a historical concern over the development of arthrofibrosis with early surgical intervention.

The limitations of this study include its retrospective nature and non-randomized design. This allowed the possibility of treatment selection bias. Whilst the cohorts overall are well matched in terms of activity level and follow-up duration, they remain heterogenous in terms of patient age and time from injury to surgery. We know that younger patients are at higher risk of re-injury following ACL surgery and indeed have demonstrated this to be the case within the repair cohort. Additionally, the study is underpowered as a sample size of over 3000 patients would be required to achieve 80% power. However, this was not possible in a study like this therefore a decision was made to pragmatically present the results with the largest available cohort of ACL repair patients. Furthermore, the difference in time from injury to surgery may have led to a reduction in activity levels in the ACL reconstruction group. These are major limitations of this comparison and a well-matched or preferably randomised controlled trial with appropriate sample size comparing rates of secondary surgery after ACL repair and reconstruction for acute proximal ACL ruptures would be required to provide further evidence.

## Conclusion

In conclusion, this study demonstrates equivalent rates of secondary surgery when comparing hamstring autograft ACL reconstruction to ACL repair with suture tape augmentation. Therefore, ACL repair with suture tape augmentation may be considered a safe and reliable alternative to ACL reconstruction in cases of acute proximal ACL ruptures.
